# Anti-Apoptosis Therapy for Meniscal Avascular Zone Repair: A Proof-of-Concept Study in a Lapine Model

**DOI:** 10.3390/bioengineering10121422

**Published:** 2023-12-14

**Authors:** Wenqiang Yan, Yue Wu, Fengyuan Zhao, Ruilan Dai, Yunan Zhou, Dingge Liu, Jin Cheng, Xiaoqing Hu, Yingfang Ao

**Affiliations:** 1 Department of Sports Medicine, Peking University Third Hospital, Institute of Sports Medicine of Peking University, Beijing 100191, China; 18752009890@163.com (W.Y.); wuyue6063@163.com (Y.W.); mickeyzhaofy@163.com (F.Z.); haharuilan@126.com (R.D.); zhouyunan66@163.com (Y.Z.); ldgog023@163.com (D.L.); chengjin@bjmu.edu.cn (J.C.); 2 Beijing Key Laboratory of Sports Injuries, Beijing 100191, China; 3 Engineering Research Center of Sports Trauma Treatment Technology and Devices, Ministry of Education, Beijing 100191, China

**Keywords:** meniscal tear, apoptosis, meniscal repair, caspases inhibitor, z-vad-fmk

## Abstract

In the present study, 24 rabbits were firstly used to evaluate the apoptosis index and matrix degeneration after untreated adult meniscal tears. Vertical tears (0.25 cm in length) were prepared in the avascular zone of the anterior horn. Specimens were harvested at 1, 3, 6, 12 weeks postoperatively. The apoptosis index around tear sites stayed at a high level throughout the whole follow-up period. The depletion of glycosaminoglycans (GAG) and aggrecan at the tear site was observed, while the deposition of COL I and COL II was not affected, even at the last follow-up of 12 weeks after operation. The expression of SOX9 decreased significantly; no cellularity was observed at the wound interface at all timepoints. Secondly, another 20 rabbits were included to evaluate the effects of anti-apoptosis therapy on rescuing meniscal cells and enhancing meniscus repair. Longitudinal vertical tears (0.5 cm in length) were made in the meniscal avascular body. Tears were repaired by the inside-out suture technique, or repaired with sutures in addition to fibrin gel and blank silica nanoparticles, or silica nanoparticles encapsulating apoptosis inhibitors (z-vad-fmk). Samples were harvested at 12 months postoperatively. We found the locally administered z-vad-fmk agent at the wound interface significantly alleviated meniscal cell apoptosis and matrix degradation, and enhanced meniscal repair in the avascular zone at 12 months after operation. Thus, local administration of caspase inhibitors (z-vad-fmk) is a promising therapeutic strategy for alleviating meniscal cell loss and enhancing meniscal repair after adult meniscal tears in the avascular zone.

## 1. Introduction

The wedge-shaped meniscal tissue between the femur and tibia plays an important role in load bearing, load transmission, shock absorption, and joint lubrication in the knee joint [1]. Meniscal damage has been shown to be one of the most common orthopedic traumas in the United States. Approximately, 850,000 operations were performed in meniscus per year, constituting 10% to 20% of all orthopedic surgeries [2,3]. The reported incidence in the general population was 0.6 to 0.7 per 1000 person years [4]. In particular, the overall incidence of meniscal injuries is 5.1 per 100,000 athlete exposures in high school athletes, with lower incidence of 3/100,000 during practice sessions and a higher incidence of 12/100,000 during games and competitions [5]. However, due to the limited healing capacity of meniscal tissue, especially in the avascular zone, meniscectomy still remains the prevailing treatment option, which causes secondary knee joint degeneration [1]. Therefore, seeking new methods to save the native meniscus is the pursuit of surgeons in the field of orthopedic and sports medicine.

Apoptosis is programmed cell death that leads to the removal of apoptotic bodies by phagocytes [6]. The critical role of apoptosis is to eliminate harmful or undesirable cells without inducing any inflammatory response, in contrast to the necrosis process in which cell contents are released by promoting an inflammatory response [7,8]. Previous studies have shown the presence of apoptotic bodies in both the meniscus and osteoarthritic cartilage, and their increase in the meniscus lacerated by trauma or degeneration [9,10,11]. Alenzi et al., investigated the positive correlation between increased apoptosis, severity of cartilage degeneration, and extracellular matrix (ECM) depletion in human osteoarthritis samples [12,13]. Moreover, the presence of apoptotic changes in injured or degenerated meniscus tissues has also been confirmed from the molecular point of view in an experimental model of osteoarthritis [14]. Finally, K. Kobayashi et al., through an alpine model study, confirmed the presence of apoptosis in meniscal tissue after partial medial meniscectomy [15].

A common characteristic of apoptosis is the activation of Caspase, a family of cysteine proteases that usually cleave after an aspartate residue in their substrates [16]. During the activation process, a great deal of cellular proteins including poly (ADP-ribose) polymerase, sterol regulatory element-binding protein (SREBP) and nuclear mitotic apparatus are cleaved by caspases [17]. Previous studies have demonstrated that inhibition of caspases could block apoptosis in many experimental models, including neurodegenerative disease, solid organ injury, and mechanically or chemically induced chondral injuries [18,19]. Thus, it was hypothesized that apoptosis inhibition could rescue meniscal fibrochondrocytes after tears, and enhance meniscal repair. The benzyloxycarbonyl-Val-Ala-Asp-fluoromethyl ketone (z-vad-fmk) is a cell-permeable broad-spectrum caspase inhibitor with no cytotoxic effects that irreversibly binds to the catalytic site of caspases [20]. Therefore, the z-vad-fmk agent was used in the present study. To enable the sustained release of z-vad-fmk molecule locally at the tear site, the hollow mesoporous silica nanoparticles (MSNs) were used to encapsulate it.

**Figure 1:**
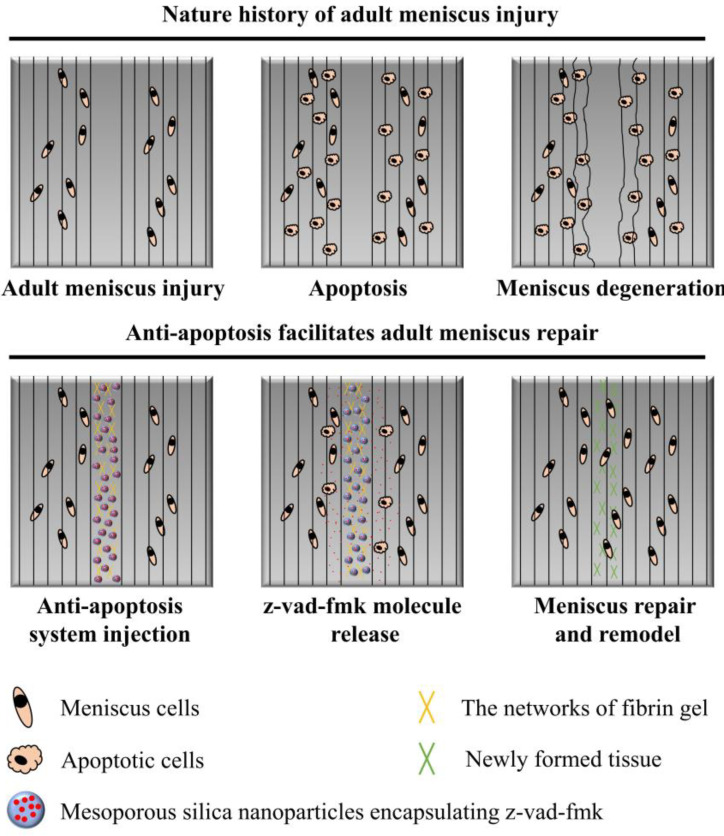
The schematics of anti-apoptosis therapy for adult meniscal repair. Apparent meniscal cell apoptosis could be observed at the tear interface following matrix degeneration. The apoptosis inhibitor (z-vad-fmk) was administered locally at the tear interface through silica nanoparticles and fibrin gel delivery system. Meniscal cell apoptosis was alleviated significantly. Finally, robust repair and integration of adult meniscal tear was achieved after remodeling.

In the present study, we firstly evaluated the severity of meniscal cell apoptosis and matrix degeneration after untreated adult meniscal tears in a lapine model. Then, the effects of apoptosis inhibition by locally administered z-vad-fmk agent on rescuing meniscal cells and enhancing meniscal repair were investigated in a longitudinal vertical tear model of the avascular zone of meniscal body. The purposes of the present study included: (1) evaluation of the severity of meniscal cell apoptosis and matrix degeneration after untreated adult meniscal tears; (2) evaluation of the effects of local administration of apoptosis inhibitor (z-vad-fmk) at the wound interface on rescuing meniscal cells and enhancing repair after tears in avascular zone. It was hypothesized that (1) adult meniscal tears caused severe meniscal cell apoptosis and matrix degeneration, and (2) local administration of apoptosis inhibitor (z-vad-fmk) at the wound interface rescued meniscal cells and enhanced meniscal repair. A schematic of the meniscal repair process is demonstrated in Figure 1. The abbreviations used in the present study are summarized in Table 1.

## 2. Materials and Methods

### 2.1. Study Design

This study was approved by the Institutional Laboratory Animal Ethics Committee of Peking University Third Hospital (approval code: A2021154, Beijing) and all experimental procedures were performed in accordance with the National Institutes of Health Guide for the Care and Use of Laboratory Animals. The present study included 44 mature male New Zealand white rabbits. For the first part of animal study, 24 rabbits were used to evaluate the presence of apoptosis and meniscal matrix degeneration after untreated meniscal tears in the avascular zone. The oblique vertical full-thickness tear (0.25 cm in length) was prepared in the avascular zone of the anterior horn of medial meniscus in right knees. Left knees were used for native control group. The specimens were harvested at 1, 3, 6, and 12 weeks postoperatively. The sample size of each group was 6. For the second part of animal study, the remaining 20 rabbits were used for subsequent evaluation of anti-apoptosis therapy on meniscal repair. The longitudinal vertical tears (0.5 cm in length) were made in the medial meniscal avascular body of both knees. The tears were repaired by the inside-out suture technique (suture group), or repaired with suture in addition to fibrin gel and blank silica nanoparticles (suture + fibrin + MSNs group), or silica nanoparticles encapsulating z-vad-fmk (suture + fibrin + MSNs + z-vad-fmk group). Each group contained five rabbits. The remaining five rabbits were used as the native control group. The knee samples were harvested at 12 months after operation. Six knee samples of each group were selected for subsequent assessment, and extra samples were reserved. We performed macroscopic and histological analyses to evaluate the response and degree of repair. The resources of antibodies, chemicals, and software used in the present study are summarized in Table 2.

### 2.2. Surgical Procedure

Animals were injected with xylazine hydrochloride (2 mg/kg) for anesthesia. After standard skin preparation and disinfection, the standard medial parapatellar approach was used. The patella was laterally dislocated. For the first part of the animal study, the tears were made in the avascular zone of the anterior horn of the medial meniscus in right knees measuring 2.5 mm in length and 1.5 mm in rim width. For the second part of the animal study, to expose the meniscal body adequately, the medial collateral ligament was transected, and then the tibia was rotated laterally. A longitudinal vertical tear (0.5 cm in length) was made in the avascular body of the medial meniscus using a scalpel. For the suture group, the wound interface was repaired with a 5–0 suture through the inside-out technique. For the suture + fibrin + MSNs group, after injecting a mixture of fibrin gel and blank MSNs (0.1 mL in total) into the wound interface, the suture was knotted to close the interface. For the suture + fibrin + MSNs + z-vad-fmk group, after injecting a mixture of fibrin gel and MSNs containing z-vad-fmk (0.1 mL in total) into the wound interface, the suture was knotted to close the interface. The medial collateral ligament was repaired using a 2–0 suture. The incision was closed through a continuous suture. The animals were injected with penicillin sodium for one week to prevent infection and were allowed to move freely in a comfortable environment.

### 2.3. Fabrication of z-Vad-Fmk Encapsulated Aminated MSNs

The commercial aminated MSNs were purchased form Nanjing Jike Biotechnology company (Nanjing, China). Firstly, the aminated MSNs were suspended in PBS solution. The morphology, particle size, and pore size of aminated MSNs were evaluated by transmission electron microscopy (FEI Tecnai F20, FEI company, Hillsboro, OR, USA). Secondly, to evaluate the zeta potential of aminated MSNs and z-vad-fmk solution at pH 7.0, the silica nanoparticles were suspended in PBS solution, the z-vad-fmk agent was firstly dissolved in DMSO solution and then diluted with PBS solution; finally, the zeta potential was evaluated (Zetasizer Nano ZS90, Malvern, UK).

For the fabrication of z-vad-fmk containing aminated MSNs, 5 mg z-vad-fmk agent was dissolved in 1 mL DMSO solution and then diluted with 9 mL PBS solution (0.5 mg/mL). Then, 50 mg aminated MSNs (5 mg/mL) was added to the prepared z-vad-fmk solution and then stirred (200 rpm) for 24 h at room temperature. Afterwards, the z-vad-fmk-encapsulated aminated MSNs were collected by centrifugation (12,000 rpm, 10 min). The commercial fibrin gel (Shanghai RAAS company, Shanghai, China) containing fibrinogen and thrombin was used to suspend aminated MSNs containing z-vad-fmk for subsequent use. For each tear, a total of 100 µL of the mixture containing 50 µL fibrinogen solution and 50 µL thrombin solution was injected into the wound interface.

### 2.4. Sample Collection and Processing

For the first part of animal study, the samples were harvested at 1, 3, 6, and 12 weeks. After 4% paraformaldehyde fixation for 2 days, the samples were embedded in paraffin. The 3 µm thick sections were prepared using microtome (Leica, Germany). Safranin O–fast green and toluidine blue staining were used to assess the deposition of glycosaminoglycans (GAG). The contents of type I collagen (COL I), type II collagen (COL II), and aggrecan were evaluated by immunohistochemical (IHC) staining. The apoptosis was evaluated by TUNEL assay. The expression of Sry-type HMG-box 9 (SOX9) meniscal cells was evaluated by immunofluorescence. For the second part of the animal study, knee samples were collected at 12 months after operation. After removing extra soft tissues, the distal femur was separated, while maintaining the menisci and tibia. The photographs were taken on the medial menisci, medial femoral condyle (MFC), and medial tibial plateau (MTP) for macroscopic evaluation. After 4% paraformaldehyde fixation for 2 days, the samples were embedded in paraffin. The 3 µm thick sections were prepared using microtome (Leica, Germany). The sections of repaired menisci were stained with hematoxylin–eosin (HE), safranin O–fast green, and immunofluorescent stains for COL I, COL II, aggrecan, SOX9, lysyl oxidase (LOX), and lysyl hydroxylase (LH2). The apoptosis at the tear interface was evaluated by TUNEL assay. The slices stained with H&E and safranin O–fast green were scanned using a digital slide scanner (NanoZoomer, Hamamatsu company, Japan). The stained slices of collagens, SOX9, LOX, LH2 and apoptosis were scanned using a confocal microscope (Leica, Germany). The degree of meniscal repair was quantified by meniscal repair scoring [21] and summarized in Table 3.

To evaluate the degree of cartilage degeneration, the MFC and MTP were fixed with 4% paraformaldehyde for 2 days; the samples were embedded in paraffin after decalcification with hydrochloric acid for 3 days. The 3 µm thick sections were prepared. The cartilage sections were stained with H&E and safranin O–fast green. The slices were scanned by a digital slide scanner (NanoZoomer, Hamamatsu). The degree of cartilage degeneration was quantified by an osteoarthritis cartilage histopathology assessment (Osteoarthritis Research Society International [OARSI] system) [22].

### 2.5. Apoptosis Tests

The apoptosis examination was completed according to the manufacture’s protocols (DeadEnd™ Fluorometric TUNEL System, G3250, Promega, USA). Briefly, the slices were permeabilized with 20 ug/mL proteinase K solution, and then processed with equilibration buffer. Then, the slices were processed with fluorescein-12-dUTP for one hour at 37 °C. Finally, the nucleus was stained with DAPI and then mounted with the relative medium. The slices were observed with a confocal fluorescence microscope (TCS-SP8, Leica, Germany). The apoptotic cells demonstrated green fluorescence, and the apoptosis index was calculated as follows: cells demonstrating green fluorescence divided by total cells within the region of interest (ROI).

### 2.6. Tissue Immunofluorescence and Immunohistochemical Staining

For tissue immunofluorescence, the sections were immersed in xylene and graded ethanol to deparaffinize and regain water. The heat-induced antigen retrieval was completed using pH 6.0 citric acid for 20 min. The nonspecific protein binding was blocked using goat serum (AR0009, Boster company, Wuhan, China) for 1 h at room temperature. The sections were then incubated with the corresponding primary antibodies for 2 h at room temperature, and subsequently incubated with the corresponding secondary antibodies for 1 h at room temperature, followed by DAPI incubation. Finally, the confocal microscope (Leica, Germany) was sued to capture the immunofluorescence. For tissue immunohistochemistry, the operation before the secondary antibodies’ incubation was identical to tissue immunofluorescence. The corresponding secondary antibodies containing HRP were used. The color was developed by using a diaminobenzidine (DAB) substrate kit. Finally, the images were captured with a digital slide scanner (NanoZoomer, Hamamatsu company, Japan). For semi-quantitative analysis, the integrated intensity of the corresponding target was evaluated with Image J software (version 1.8.0, US National Institutes of Health, USA).

### 2.7. Statistical Analysis

A priori power analysis was performed using G*Power software (G*Power, version 3.1.9.2) to calculate the appropriate sample size. At the α level of 0.05, a sample size of six was necessary for each group to achieve a power of 0.8 and an effect size of 0.8. The data are expressed as mean values with standard deviations (SD). For the first part of animal study, a two-way ANOVA with a Bonferroni multiple comparisons test was applied for pairwise comparison of the matrix deposition between the tear and control groups. For the second part of the animal study, an ordinary one-way ANOVA with a Bonferroni multiple comparisons test was applied. All statistical analyses were completed in GraphPad Prism software (GraphPad Software, version 8.0.1). A *p* value < 0.05 was considered statistically significant.

## 3. Results

### 3.1. Apoptosis Examination after Untreated Adult Meniscal Tears

**Figure 2:**
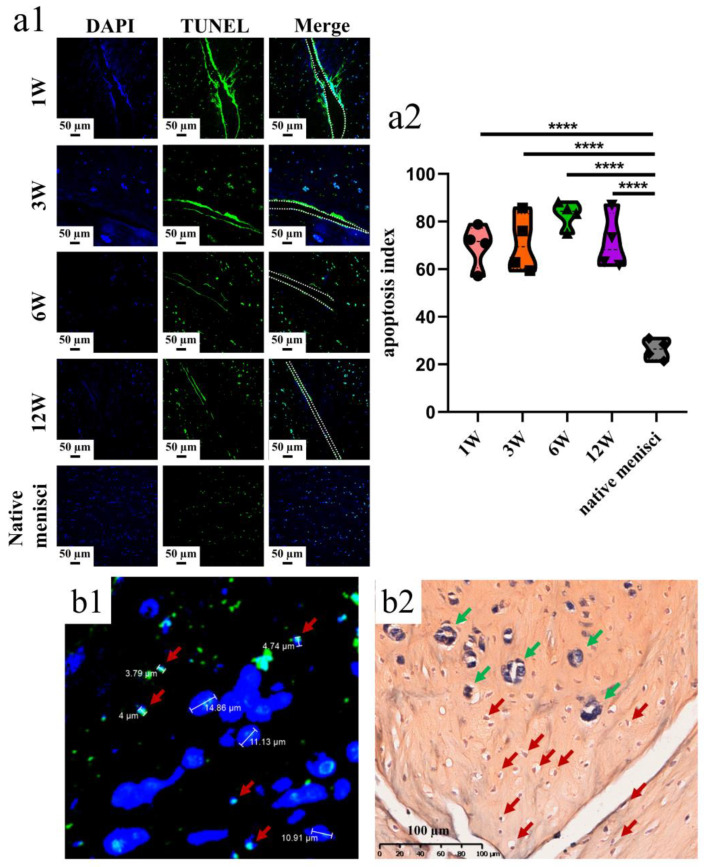
The presence of meniscal cell apoptosis at the tear site after untreated adult meniscal tears in the avascular zone. (**a1**) meniscal cell apoptosis at the tear site at different timepoints; green fluorescence indicates apoptotic cells, and W represents week; (**a2**) violin plots of apoptosis index, *n* = 4, one-way ANOVA, ****, *p* < 0.0001; (**b1**) pyknosis reflected by TUNEL assay and DAPI. Red arrows represent apoptotic cells; (**b2**) pyknosis reflected by hematoxylin. Red arrows represent apoptotic cells, and green arrows represent relative normal meniscal fibrochondrocytes.

Apoptotic cells were dispersed along the tear edge after adult meniscal tears (Figure 2(a1)). The apoptosis index was 69.81 ± 9.11%, 71.00 ± 12.14%, 82.97 ± 5.68%, 71.29 ± 11.66% at 1, 3, 6, 12 weeks, respectively after operation. The apoptosis index of the normal meniscus was 26.30 ± 4.23% (Figure 2(a2)). The typical apoptotic characteristic phenomenon of pyknosis (the condensation of chromatin) was observed in the TUNEL apoptosis examination. The size of the relative normal meniscal cell nuclei was approximately 10 µm, while the size of the apoptotic cell nuclei was around 5 µm (Figure 2(b1)). Moreover, after safranin O–fast green staining, the nuclei demonstrated weak staining of hematoxylin and a small size, which indicated apoptosis (Figure 2(b2)).

### 3.2. Meniscal Matrix Degradation after Untreated Adult Meniscal Tears

**Figure 3:**
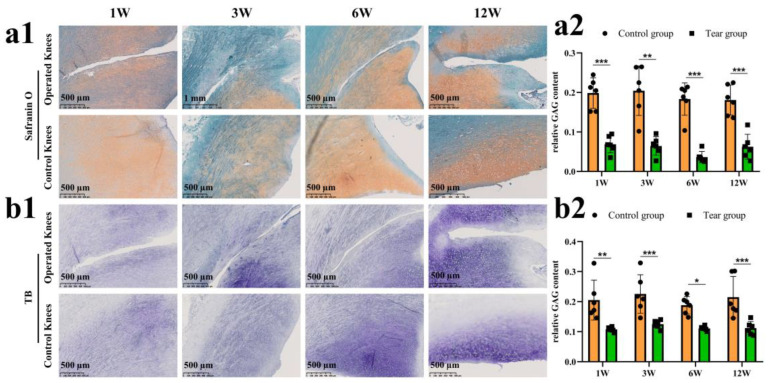
The evaluation of proteoglycan degradation after untreated adult meniscal tears. (**a1**) proteoglycan degradation evaluated by safranin O–fast green staining; (**a2**) semi-quantitative analysis of GAG content reflected by safranin O–fast green staining, *n* = 6, and a pairwise comparison test of two-way ANOVA, **, *p* < 0.01, ***, *p* < 0.0005); (**b1**) proteoglycan degradation evaluated by toluidine blue staining; (**b2**), semi-quantitative analysis of GAG content reflected by toluidine blue staining, *n* = 6, and a pairwise comparison test of two-way ANOVA, *, *p* < 0.05, **, *p* < 0.01, ***, *p* < 0.0005), the circle symbol represents control group, the cube symbol represents tear group.

Safranin O–fast green and toluidine blue staining was used to evaluate GAG deposition. The GAG deposition around the tear site was inferior compared to the controls. Even at the last follow up, no recovery of GAG deposition was found. The GAG depletion zone enlarged gradually (Figure 3(a1)). The semiquantitative analysis showed consistent results, as demonstrated in safranin O–fast green staining. Significant differences in relative GAG content could be observed between the tear and control groups at 1 week (mean, 0.07 versus 0.20, *p* < 0.0005), 3 weeks (mean, 0.06 versus 0.20, *p* < 0.01), 6 weeks (mean, 0.04 versus 0.18, *p* < 0.0005) and 12 weeks (mean, 0.06 versus 0.18, *p* < 0.0005) postoperatively (Figure 3(a2)). Similar results were also demonstrated in the toluidine blue staining (Figure 3(b1,b2)).

**Figure 4:**
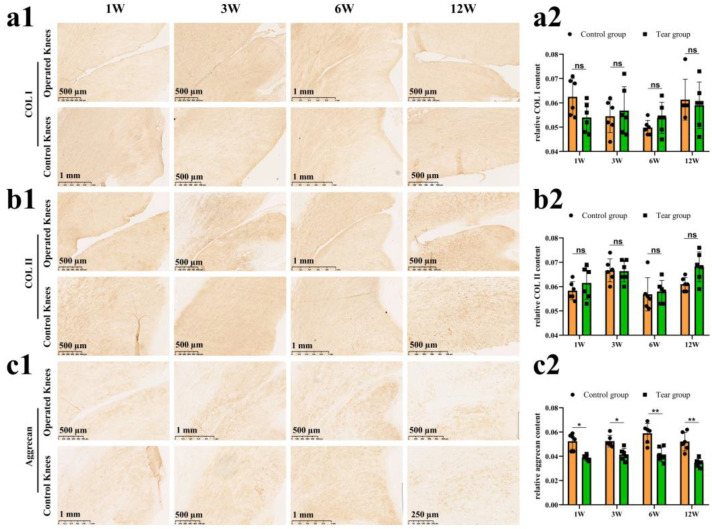
The evaluation of COL I, COL II and aggrecan after untreated adult meniscal tears. (**a1**) IHC staining for COL I; (**a2**) semi-quantitative analysis of COL I content, *n* = 6, and a pairwise comparison test of two-way ANOVA. ns refers to no significant difference; (**b1**) IHC staining for COL II; (**b2**) semi-quantitative analysis of COL II content, *n* = 6, and a pairwise comparison test of two-way ANOVA; (**c1**) IHC staining for aggrecan; (**c2**) semi-quantitative analysis of aggrecan content, *n* = 6, and a pairwise comparison test of two-way ANOVA). *, *p* < 0.05, **, *p* < 0.01, ns, no significance), the circle symbol represents control group, the cube symbol represents tear group.

**Figure 5:**
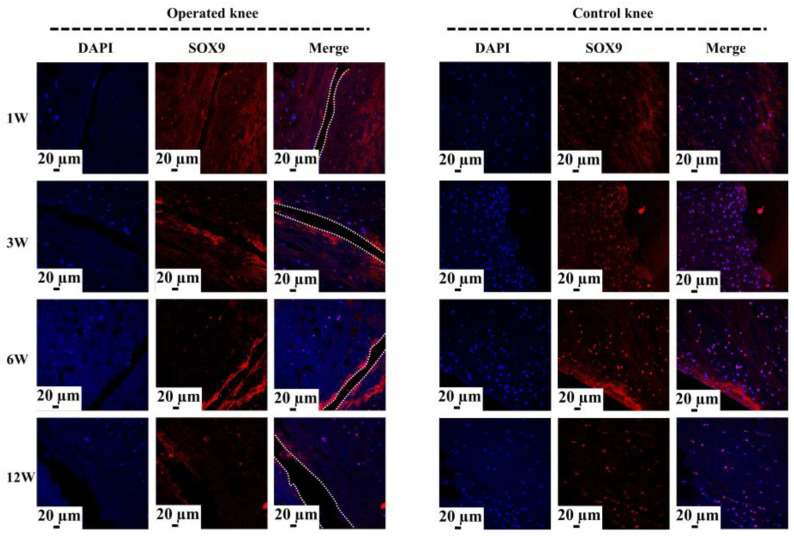
The analysis of SOX9 expression at the tear site after untreated adult meniscal tears. The white dotted lines indicate the tear edge. The SOX9 positively expressed cells have a pink color in the merged images.

The COL I content was assessed via an IHC test (Figure 4(a1)). There was no significant difference in relative COL I content between the control and tear groups at 1 week (mean, 0.06 vs. 0.05, ns), 3 weeks (mean, 0.05 vs. 0.06, ns), 6 weeks (mean, 0.05 vs. 0.05, ns), and 12 weeks (mean, 0.06 vs. 0.06, ns) postoperatively (Figure 4(a2)). The COL II content was assessed by an IHC test (Figure 4(b1)). There was no significant difference in relative COL II content between the control and tear groups at 1 week (mean, 0.06 vs. 0.06, ns), 3 weeks (mean, 0.07 vs. 0.07, ns), 6 weeks (mean, 0.06 vs. 0.06, ns), and 12 weeks (mean, 0.06 vs. 0.07, ns) postoperatively (Figure 4(b2)). The aggrecan deposition was evaluated by the IHC. Reduction in aggrecan deposition, assessed by IHC staining, around the tear site has been demonstrated in all torn samples (Figure 4(c1)). Significant differences in relative aggrecan content could be observed between the control and tear group at 1 week (0.05 vs. 0.04, *p* < 0.05), 3 weeks (0.05 vs. 0.04, *p* <0.05), 6 weeks (0.06 vs. 0.04, *p* < 0.01), and 12 weeks (0.05 vs. 0.03, *p* < 0.01) postoperatively (Figure 4(c2)). The expression of SOX9 in meniscal cells was evaluated by immunofluorescence. A DAPI negatively stained zone with a width of approximate 100 μm was present at the tear site at 1 week postoperatively, and the SOX9 expression disappeared, when compared to the contralateral native menisci. Moreover, no cellularization was observed at the tear edge at 3, 6, and 12 weeks postoperatively. In the control groups, SOX9 was strongly expressed in meniscal cells (Figure 5).

### 3.3. Sustained Release of z-Vad-Fmk Alleviated Apoptosis and Enhanced Adult Meniscal Repair

**Figure 6:**
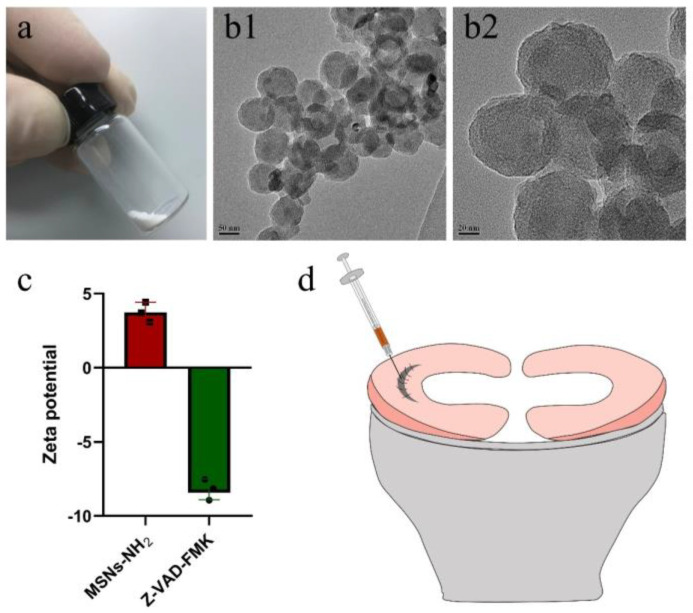
The characterization of aminated mesoporous silica nanoparticles (MSNs–NH_2_) and z-vad-fmk. The preparation of meniscal tears in a lapine model. (**a**) MSNs–NH_2_ powder; (**b1**) transmission electron microscopic evaluation of MSNs–NH_2_ at low magnification; (**b2**) transmission electron microscopic evaluation of MSNs–NH_2_ at high magnification; (**c**) the zeta potential of MSNs–NH_2_ and z-vad-fmk solution at pH = 7.0, *n* = 3, the cube symbol represents MSNs–NH_2_, the circle symbol represents z-vad-fmk; (**d**) the preparation and repair of a longitudinal vertical tear in the avascular body of the medial meniscus. The fibrin gel containing MSNs–NH_2_–z-vad-fmk is injected into the wound interface, then the suture was knotted to close the tear.

**Figure 7:**
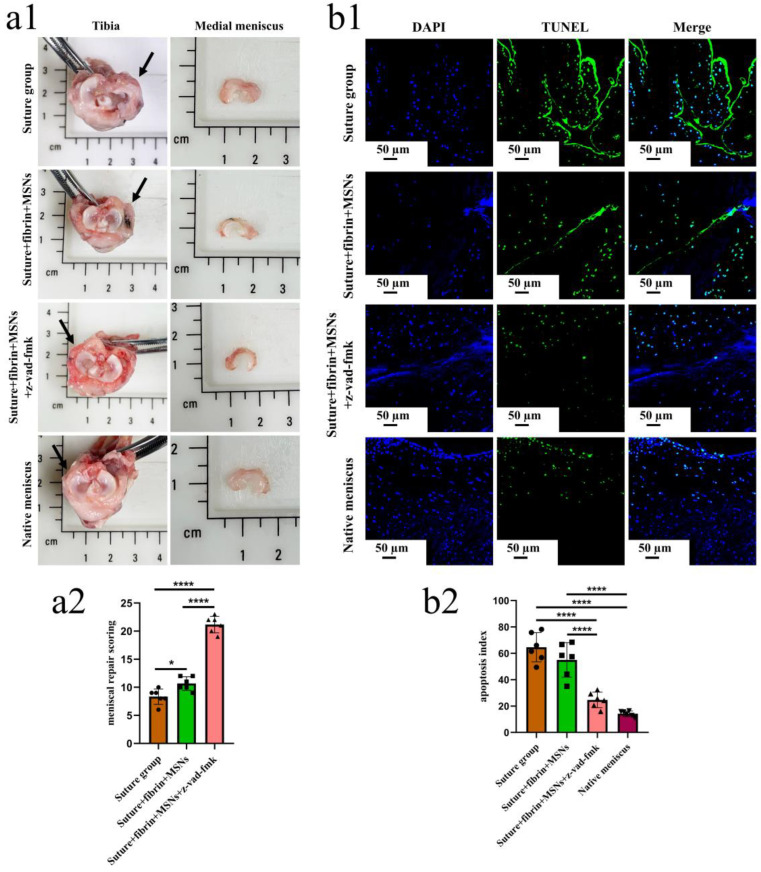
The macroscopic evaluation of meniscal repair and meniscal repair scoring. The apoptosis evaluation around the tear edge in the repaired menisci. (**a1**), the macroscopic analysis of meniscal repair; black arrows represent the medial meniscus; (**a2**) Meniscal repair scoring of different groups, *n* = 6, one-way ANOVA, *, *p* < 0.05, ****, *p* < 0.0001, the circle symbol represents the suture group, the cube symbol represents the suture + fibrin + MSNs group, the triangle symbol represents the suture + fibrin + MSNs + z-vad-fmk group; (**b1**), the presence of apoptosis around the tear site evaluated by TUNEL assay in the repaired menisci; (**b2**), the apoptosis index at the tear site in different groups, *n* = 6, one-way ANOVA, ****, *p* < 0.0001, the circle symbol represents the suture group, the cube symbol represents the suture + fibrin + MSNs group, the upper triangle symbol represents the suture + fibrin + MSNs + z-vad-fmk group, the lower triangle symbol represents the native meniscus.

To enable the sustained release of z-vad-fmk, the aminated hollow mesoporous silica nanoparticles (MSNs-NH_2_) with a mean particle size of 100 nm and mean pore size of 2–3 nm was used to encapsulate it (Figure 6(a,b1,b2)). At pH 7.0, the surfaces of MSNs-NH_2_ mainly contained positive charges, while the z-vad-fmk solution mainly contained negative charges (Figure 6c). Firstly, the positive and negative charge interactions caused z-vad-fmk molecules to adhere to the surface of MSNs-NH_2_. Secondly, the mesoporous silica nanoparticle was hollow, which could encapsulate z-vad-fmk molecule. After encapsulation, the MSNs-NH_2_-z-vad-fmk was mixed with fibrin gel for subsequent injection. In the present study, a longitudinal vertical tear (0.5 cm in length) was made in the medial meniscal avascular body of the lapine model. The tears were repaired by the inside-out suture technique (suture group), or repaired with sutures in addition to fibrin gel and blank silica nanoparticles (suture + fibrin + MSNs group), or silica nanoparticles containing z-vad-fmk (suture + fibrin + MSNs + z-vad-fmk group) (Figure 6d). At 12 months after operation, all knee samples were harvested for macroscopic and histological evaluations. The sutures used for repair were removed. Macroscopically, the wound gap in the suture group could be clearly identified. There was no new tissue at the wound edge. Moreover, obvious degeneration was observed along the tear edge. Similar findings were observed in the suture + fibrin + MSNs group. In the suture + fibrin + MSNs + z-vad-fmk group, meniscal tears could not be identified from a macroscopic view (Figure 7(a1)). From the perspective of meniscal repair scoring, compared to the suture group (mean repair score, 8.33) or the suture + fibrin + MSNs group (mean repair score, 10.67), the suture + fibrin + MSNs + z-vad-fmk group possessed the highest repair scores (mean repair score, 21.17) (Figure 7(a2)). In order to evaluate the anti-apoptosis effect of z-vad-fmk at the tear edge, a TUNEL apoptosis assay was used for histological assessment. In the suture group and suture + fibrin + MSNs groups, large quantities of apoptotic cells could be observed around the tear edges, while in the suture + fibrin + MSNs + z-vad-fmk group, the numbers of apoptotic cells decreased significantly around the tear edges (Figure 7(b1)). The apoptosis indexes at the tear edge in the suture group (mean, 64.64%) and the suture + fibrin + MSNs group (mean, 55.04%) were significantly higher than those of the z-vad-fmk treatment group (mean, 24.70%) and native menisci (mean, 14.19%) (Figure 7(b2)).

**Figure 8:**
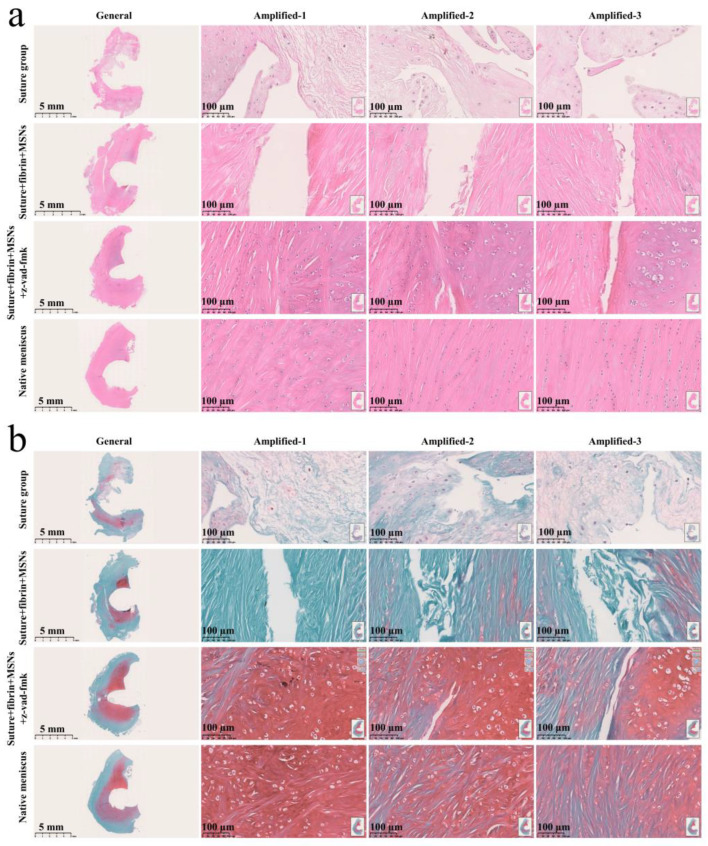
Histological analysis of meniscal repair in different groups. (**a**) H&E staining; (**b**) safranin O–fast green staining. In H&E staining, the meniscus matrix is stained with red color, and the nucleus of the meniscus cell is stained with blue color. In safranin O–fast green staining, the GAG matrix is stained with red color. GAG represents glycosaminoglycans.

**Figure 9:**
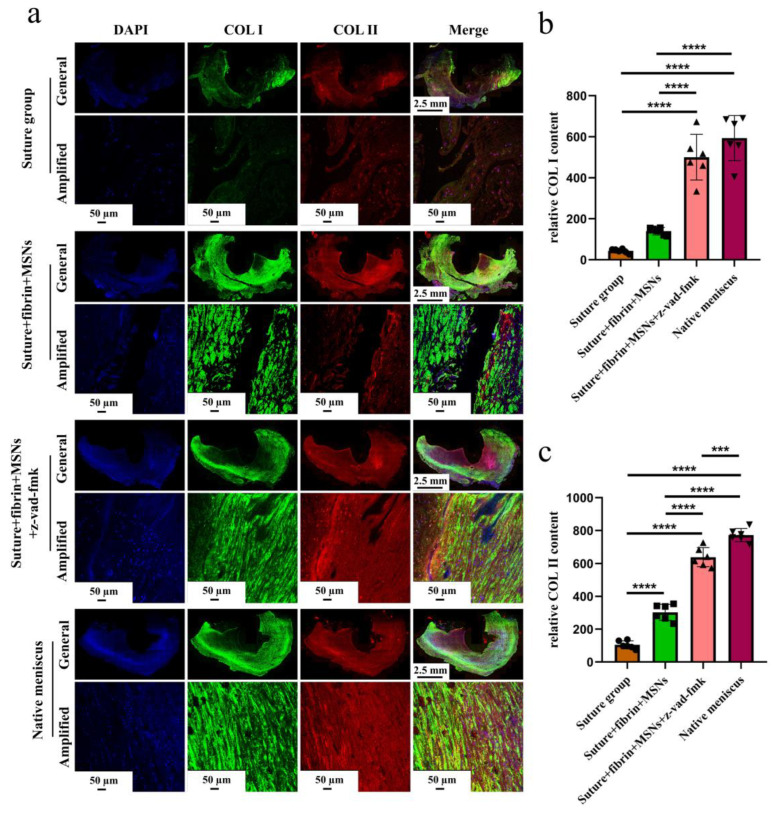
The evaluation of COL I and COL II deposition in repaired menisci. (**a**) Immunofluorescent co-staining of COL I and COL II in the repaired menisci and native menisci; (**b**) semi-quantitative analysis of COL I content in different groups, *n* = 6, one-way ANOVA; (**c**) semi-quantitative analysis of COL II content in different groups, *n* = 6, one-way ANOVA. ***, *p* < 0.0005, ****, *p* < 0.0001, the circle symbol represents the suture group, the cube symbol represents the suture + fibrin + MSNs group, the upper triangle symbol represents the suture + fibrin + MSNs + z-vad-fmk group, the lower triangle symbol represents the native meniscus.

**Figure 10:**
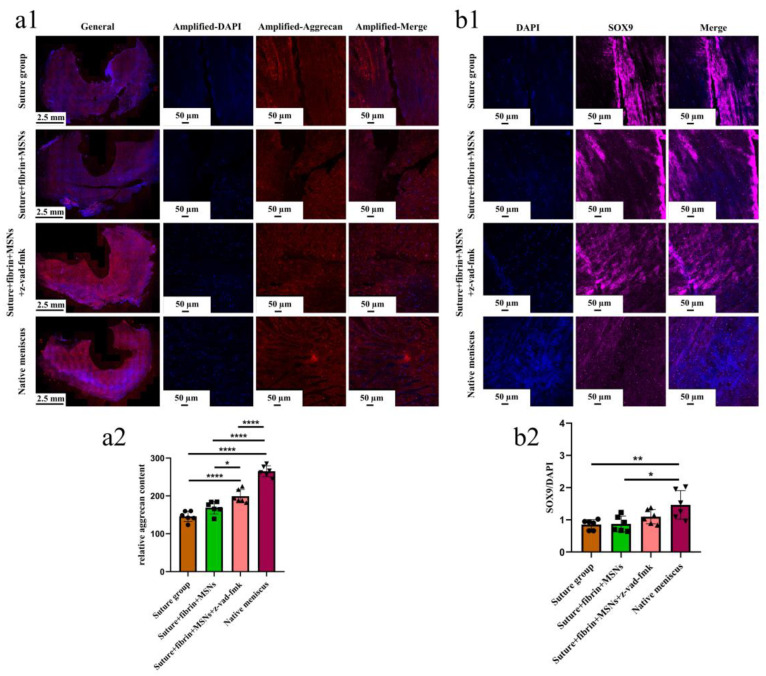
The evaluation of aggrecan and SOX9 expression in repaired menisci. (**a1**) immunofluorescent staining of aggrecan; (**a2**) the semi-quantitative analysis of aggrecan content in different groups, *n* = 6, one-way ANOVA; (**b1**) the immunofluorescent staining of SOX9; (**b2**) the semi-quantitative analysis of SOX9 expression in different groups, *n* = 6, one-way ANOVA. *, *p* < 0.05, **, *p* < 0.01, ****, *p* < 0.0001, the circle symbol represents the suture group, the cube symbol represents the suture + fibrin + MSNs group, the upper triangle symbol represents the suture + fibrin + MSNs + z-vad-fmk group, the lower triangle symbol represents the native meniscus.

**Figure 11:**
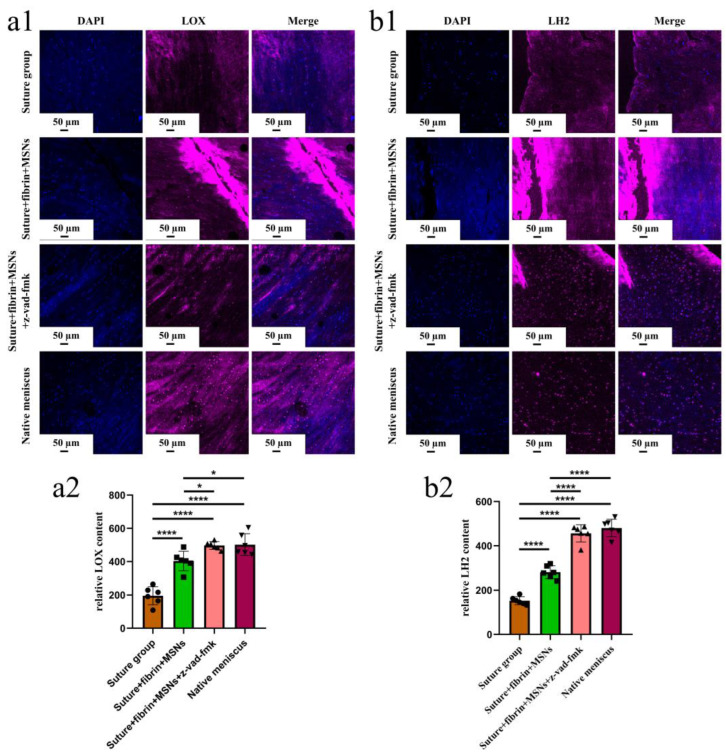
The evaluation of LOX and LH2 in the repaired and native menisci. (**a1**) the immunofluorescent staining of LOX; (**a2**) semi-quantitative analysis of LOX expression in different groups, *n* = 6, one-way ANOVA; (**b1**) immunofluorescent staining of LH2; (**b2**) semi-quantitative analysis of LH2 expression in different groups, *n* = 6, one-way ANOVA. *, *p* < 0.05, ****, *p* < 0.0001, the circle symbol represents the suture group, the cube symbol represents the suture + fibrin + MSNs group, the upper triangle symbol represents the suture + fibrin + MSNs + z-vad-fmk group, the lower triangle symbol represents the native meniscus.

**Figure 12:**
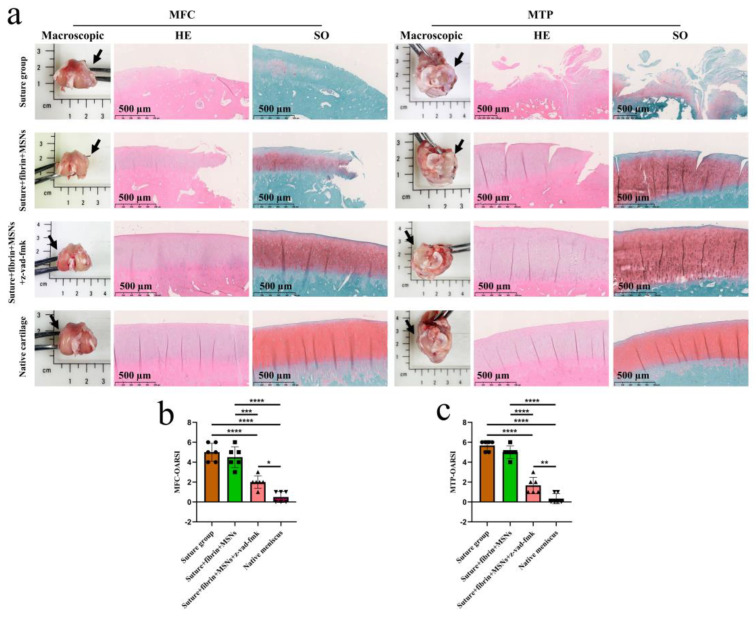
The assessment of knee joint cartilage degeneration. (**a**) macroscopic and histological analysis of cartilage degeneration in MFC and MTP. Black arrows represent the medial side. MFC, medial femoral condyle; MTP, medial tibial plateau; SO, safranin O–fast green; (**b**) semi-quantitative OARSI scores of cartilage degeneration in MFC, *n* = 6, one-way ANOVA; (**c**) semi-quantitative OARSI scores of cartilage degeneration in MTP, *n* = 6, one-way ANOVA. OARSI, osteoarthritis cartilage histopathology assessment (Osteoarthritis Research Society International [OARSI] system. *, *p* < 0.05, **, *p* < 0.01, ***, *p* < 0.0005, ****, *p* < 0.0001, the circle symbol represents the suture group, the cube symbol represents the suture + fibrin + MSNs group, the upper triangle symbol represents the suture + fibrin + MSNs + z-vad-fmk group, the lower triangle symbol represents the native meniscus.

Various findings in the histological staining of repaired menisci could be observed in different groups. For H&E staining, the wound gap in the suture group and suture + fibrin + MSNs group could be clearly observed in the avascular body of the meniscus. Obvious degeneration was observed at the wound edge, demonstrating decreased cellularity and disorganized collagen bundles compared to native meniscal tissue, while the suture + fibrin + MSNs + z-vad-fmk group demonstrated a robust healing response with superior interface integration, increased cellularity, and organized collagen arrangement (Figure 8a). Safranin O–fast green staining was used to evaluate the content of proteoglycans. In the suture group and suture + fibrin + MSNs group, the contents of proteoglycans in the wound interface decreased significantly, while abundant deposition of proteoglycans was maintained in suture + fibrin + MSNs + z-vad-fmk group (Figure 8b). The deposition of COL I, COL II, and aggrecan was evaluated by immunofluorescence. In the suture group and suture + fibrin + MSNs group, the contents of COL I, COL II and aggrecan decreased significantly. However, the contents of COL I, COL II and aggrecan in the suture + fibrin + MSNs + z-vad-fmk group were maintained, approximating those of native menisci (Figure 9 and Figure 10(a1,a2)). Meanwhile, the expression of transcriptional factor-SOX9 was evaluated by immunofluorescence. In the suture + fibrin + MSNs + z-vad-fmk group, the reparative cells at the wound interface demonstrated more robust expression of SOX9, like native meniscal fibrochondrocytes, compared to that of the suture group and suture + fibrin + MSNs groups (Figure 10(b1,b2)). The lysyl oxidase (LOX) and lysyl hydroxylase-2 (LH2) were responsible for collagen crosslinking within the meniscus. Compared to the suture group and suture + fibrin + MSNs group, the suture + fibrin + MSNs + z-vad-fmk group possessed the strongest expression of LOX and LH2, resembling that of native meniscal tissue (Figure 11). The knee joint cartilage status after tears and repair was evaluated at 12 months postoperatively, including the medial femoral condyle and medial tibial plateau. Apparent chondral injuries, demonstrating cartilage erosions, uneven surface, and cartilage matrix degradation, were identified in the suture group and suture + fibrin + MSNs group. However, the suture + fibrin + MSNs + z-vad-fmk group demonstrated an even and intact cartilage surface, as well as rich cartilage matrix deposition resembling that of native cartilage (Figure 12a). The degree of cartilage degeneration was scored using the OARSI system. Compared with the suture group and suture + fibrin + MSNs group, the suture + fibrin + MSNs + z-vad-fmk group possessed the lowest OARSI scores in MFC and MTP, despite the OARSI score of all groups being higher than that of the native meniscal group (Figure 12b,c).

## 4. Discussion

Multiple previous studies have indicated that acute or degenerated meniscal injuries induce high levels of meniscal cell apoptosis. Mustafa Uysal et al. [11] investigated apoptotic changes in 24 human meniscal specimens after degenerative or traumatic meniscal tears, and 14 normal human meniscal samples harvested from cadavers. They concluded that the torn meniscal tissues caused by either degeneration or trauma had a higher apoptosis index compared to normal meniscal tissues. Another study reported an increase in apoptosis in the inner avascular portion of meniscus in an experimental osteoarthritis model induced by anterior cruciate ligament transection in a lapine model [9]. This finding was not surprising, because multiple essential mediators of chondrocytes’ apoptosis were presented, including mechanical imbalance, disruption of the meniscus structure, inflammatory cells, proinflammatory cytokines, and so on [19,23].

Despite results in meniscus tissues, apoptosis has been demonstrated to be positively correlated with the severity of cartilage degradation and ECM depletion in human osteoarthritic cartilage specimens [12,13]. Moreover, apoptosis plays an essential role in the pathogenesis of intervertebral disc degeneration (IDD) with ECM degradation [24]. Whether chondrocyte apoptosis is a cause or a consequence of cartilage degradation in OA is hotly contested. In the present study, we observed a more prominent apoptosis phenomenon in the more severely degenerated zone of the meniscus. However, the present study could not specify whether meniscal cell apoptosis is a cause or a consequence of ECM degradation. If the apoptotic changes were considered to be the cause of ECM degradation after untreated meniscal tears, the presence of apoptosis could contribute to the destructive degeneration in meniscal tissue through at least two mechanisms: (i) As a consequence of apoptosis, the cellular components that were responsible for maintaining and remodeling the meniscal tissue decreased. (ii) The apoptotic body of membrane-enclosed units containing cellular components was a consequence of apoptosis, and maintained in the ECM. The accumulation of apoptotic bodies in pericellular or interterritorial matrices could lead to matrix damage [10]. Moreover, a recent review has indicated the role of apoptosis in extraosseous calcification [25]. If the apoptotic changes are considered to be the consequence of ECM degradation after untreated meniscal tears, ECM degradation could contribute to apoptotic changes through the following mechanism: the loss of ECM components (collagen denaturation, aggrecan, GAG degradation, or other matrix component degeneration), which disturbs cell–matrix interaction, which is critical for the survival of cells. This was supported by the phenomenon of “anchorage dependence”, highlighting that cells attached to the ECM components or each other to survive, and cells were prone to undergo apoptosis when the ECM was degraded or decreased [26]. However, no matter if apoptosis is a cause or a consequence of matrix degeneration, the inhibition of meniscal cell apoptosis is hypothesized to alleviate matrix degradation and enhance tissue repair.

In the present study, we demonstrated that the apoptosis index reached a high level in the first week after untreated meniscal tears. This early post-tear timepoint was postulated to represent a window of opportunity for treatment intervention. We found that the timely administration of caspase inhibitors (z-vad-fmk) to meniscal tears alleviated meniscal cell apoptosis and matrix depletion. Superior tear interface integration was observed after treatment with z-vad-fmk. LOX and LH2 are two common enzymes that catalyze collagen crosslinking, which determine the integrity of collagen networks [27,28]. In the present study, the expression of LOX and LH2 in meniscal cells was maintained after z-vad-fmk treatment, resembling native meniscal cells, which was critical to tear closure. Thereby, we provided a potential strategy for meniscal repair based on alleviating meniscal cell apoptosis. As programmed cell death was a normal physiological incidence in biological processes, the side effect caused by caspase inhibitors on tissue repair or phenotype was a concern in the present study. In the musculoskeletal system, programmed cell death participated in multiple pathways critical to tissue repair mechanisms [29]. Thus, it was possible that anti-apoptosis therapy through caspase inhibition would affect repair in our meniscal tear model. However, the local administration of z-vad-fmk at the tear site did not affect meniscal healing; instead, meniscal repair was enhanced robustly. Importantly, other adverse effects of caspase inhibition on the incidence of infection or the general health status of rabbits were not observed. The present study emphasized the benefits of targeted local administration of caspase inhibitors through mesoporous silica nanoparticles and fibrin gel within the knee joint cavity, which minimized the amount of drugs administered.

Some limitations still exist in the present study. Firstly, the present study only observed the anti-apoptosis effect and meniscal repair at 12 months postoperatively. The apoptosis alleviation and meniscal repair in the early phase were not investigated. Secondly, the transformation and metabolism of silica nanoparticles were not investigated. Whether the silica nanoparticles were taken up by cells, suspended within the knee joint cavity, or entered into blood circulation needs further investigation. Thirdly, the optimal z-vad-fmk doses, timing, duration of treatment, and release model in vivo, as well as long-term effects on joint function, necessitate further study. Lastly, the mechanical properties of repaired menisci were not investigated in the present study due to the deficiency in small size of rabbit menisci. We plan to carry out further study on large animals like goats and pigs, which are similar to human beings in joint size and physiology. Then, the mechanical properties will be included.

## 5. Conclusions

Meniscal tear causes severe meniscus cell apoptosis and matrix degeneration. The local administration of apoptosis inhibitors (z-vad-fmk) could alleviate meniscal cell apoptosis and enhance meniscus repair after tears in the avascular zone 12 months postoperatively in a lapine model.

## Figures and Tables

**Table 1:** Abbreviations used.

Abbreviations	Definition
z-vad-fmk	Benzyloxycarbonyl-Val-Ala-Asp-fluoromethyl ketone
GAG	Glycosaminoglycans
COL I	Type I collagen
COL II	Type II collagen
SOX9	Sry-type HMG-box 9
ECM	Extracellular matrix
caspase	Cysteinyl aspartate-specific proteases
MSNs	Mesoporous silica nanoparticles
PBS	Phosphate-buffered solution
HE	Hematoxylin-eosin
DMSO	Dimethylsulfoxide
IHC	Immunohistochemical
MFC	Medial femoral condyle
MTP	Medial tibial plateau
OARSI	Osteoarthritis Research Society International
LOX	Lysyl oxidase
LH	Lysyl hydroxylase
DAPI	4′,6-diamidino2-phenylindole
ROI	Region of interest
HRP	Horseradish peroxidase
DAB	Diaminobenzidine
SD	Standard deviation
ANOVA	Analysis of variance
ns	No significant difference
IDD	Intervertebral disc degeneration

**Table 2:** Key resources table.

Reagent or Resource	Source	Identifier
Primary antibodies		
Mouse anti collagen II monoclonal antibody	Invitrogen, Rockford, IL, USA	MA5-13026
Rabbit anti SOX9 antibody	Sigma, Darmstadt, Germany	HPA001758
Goat anti collagen I antibody	Arigo, China	ARG21965
Mouse anti aggrecan antibody	MilliporeSigma, Darmstadt, Germany	C8035
Rabbit anti LOX polyclonal antibody	Proteintech, Wuhan, China	17958-1-AP
Rabbit anti PLODA2 (LH2) antibody	Proteintech, Wuhan, China	21214-1-AP
Secondary antibodies		
Donkey anti-rabbit IgG H & L (Alexa Fluor^®^ 647)	Abcam, Cambridge, UK	ab150075
Donkey anti-mouse IgG H & L (Alexa Fluor^®^ 594)	Abcam, Cambridge, UK	ab150108
Donkey anti-goat IgG H & L (Alexa Fluor^®^ 488)	Abcam, Cambridge, UK	ab150129
Goat anti-mouse IgG H & L (HRP)	Abcam, Cambridge, UK	ab6789
Donkey anti-Goat IgG H & L (HRP)	Abcam, Cambridge, UK	ab6885
Chemicals and reagent		
z-vad-fmk	MCE, Monmouth Junction, NJ, USA	HY-16658B
TUNEL assay	Promega, Madison, MI, USA	G3250
Software		
GraphPad Prism software	GraphPad	version 8.0.1
Image J software	NIH	version 1.8.0
G*Power software	G*Power	version 3.1.9.2

**Table 3:** Scoring system for evaluation of the quality of meniscal repair tissue.

	0	1	2	3
Defect filling	No fill	<25%	25–75%	>75%
Surface	No surface	Ruptured	Fissured/fibrillated	Meniscus-like
Integration	No integration	Partial, unilateral integration	Bilateral partial or unilateral complete integration	Bilateral complete integration
Cellularity	No cells	>10 cell cluster/slide	No cell cluster/slide, Cell–ECM ratio > 0.5	Meniscus-like cell–ECM ratio
Cell morphology	No cells	<25% Meniscus-like cells	25–75% Meniscus-like cells	>75% Meniscus-like cells
Content of proteoglycan	No staining for proteoglycan	<25%	25–75%	>75%
Content of type 2 collagen	No staining for type 2 collagen	<25%	25–75%	>75%
Stability	No stability	Weak	Stable in shape	Stable to pressure and pulling stress

## Data Availability

The datasets used and/or analyzed during the current study are available from the corresponding author on reasonable request.
